# Critical behavior of a water monolayer under hydrophobic confinement

**DOI:** 10.1038/srep04440

**Published:** 2014-04-04

**Authors:** Valentino Bianco, Giancarlo Franzese

**Affiliations:** 1 Departament de Física Fonamental, Universitat de Barcelona, Martí i Franquès 1, ES-08028 Barcelona, Spain

## Abstract

The properties of water can have a strong dependence on the confinement. Here, we consider a water monolayer nanoconfined between hydrophobic parallel walls under conditions that prevent its crystallization. We investigate, by simulations of a many-body coarse-grained water model, how the properties of the liquid are affected by the confinement. We show, by studying the response functions and the correlation length and by performing finite-size scaling of the appropriate order parameter, that at low temperature the monolayer undergoes a liquid-liquid phase transition ending in a critical point in the universality class of the two-dimensional (2D) Ising model. Surprisingly, by reducing the linear size *L* of the walls, keeping the walls separation *h* constant, we find a 2D-3D crossover for the universality class of the liquid-liquid critical point for 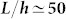, i.e. for a monolayer thickness that is small compared to its extension. This result is drastically different from what is reported for simple liquids, where the crossover occurs for 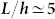, and is consistent with experimental results and atomistic simulations. We shed light on these findings showing that they are a consequence of the strong cooperativity and the low coordination number of the hydrogen bond network that characterizes water.

The study of nanoconfined water is of great interest for applications in nanotechnology and nanoscience^1^. The confinement of water in quasi-one or two dimensions (2D) is leading to the discovery of new and controversial phenomena in experiments^1^,^2^,^3^,^4^,^5^ and simulations^4^,^6^,^7^. Nanoconfinement, both in hydrophilic and hydrophobic materials, can keep water in the liquid phase at temperatures as low as 130 K at ambient pressure^2^. At these temperatures *T* and pressures *P* experiments cannot probe liquid water in the bulk, because water freezes faster then the minimum observation time of usual techniques, resulting in an experimental “no man's land”^8^. Nevertheless, new kind of experiments^9^,^10^ and numerical simulations^11^ can access this region, revealing interesting phenomena in the metastable state. In particular, Poole et al. found, by molecular dynamics simulations of supercooled water, a liquid-liquid critical point (LLCP), in the “no mans land”, at the end of a first–order liquid-liquid phase transition (LLPT) line between two metastable liquids phases with different density *ρ*: the high-density liquid (HDL) at higher *T* and *P*, and the low-density liquid (LDL) at lower *T* and *P*^11^. The presence of a LLPT is experimentally observed in other systems^12^,^13^,^14^,^15^,^16^,^17^,^18^,^19^,^20^,^21^, consistent with theoretical models fitted to water experimental data^22^,^23^,^24^, and is recovered by simulations of a number of models of water^11^,^25^,^26^,^27^,^28^,^29^,^30^,^31^ and other anomalous liquids^32^,^33^,^34^,^35^,^36^,^37^. Alternative ideas, and their differences, have been discussed^38^,^39^,^40^,^41^,^42^, and it has been debated if experiments on confined water in the “no man's land” can be a way to test these ideas^2^, motivating several theoretical works^43^.

Here, to analyze the thermodynamic properties of water in confinement we consider a water monolayer between hydrophobic walls of area *L*^2^ separated by *h* ≈ 0.5 nm (Fig. 1). Atomistic simulations^7^ show that water under these conditions does not crystallize, but arranges in a disordered liquid layer, whose projection on one of the surfaces has square symmetry, with each water molecule having four nearest neighbors (n.n.). The molecules maximize their intermolecular distance by adjusting at different heights with respect to the two walls.

We adopt a *many-body* model that reproduces water properties^31^,^40^,^44^,^45^,^46^,^47^,^48^,^49^,^50^. We simulate ~ 10^5^ state points, each with statistics of 5 × 10^6^ independent calculations, for systems with *N* = 2.5 × 10^3^, …, 1.6 × 10^5^ water molecules at constant *N*, *P* and *T*, using a cluster Monte Carlo algorithm^46^,^47^,^48^, for a wide range of *T* and *P*. All quantities are calculated in internal units, as described in the Methods section.

## Results

**Figure 2:**
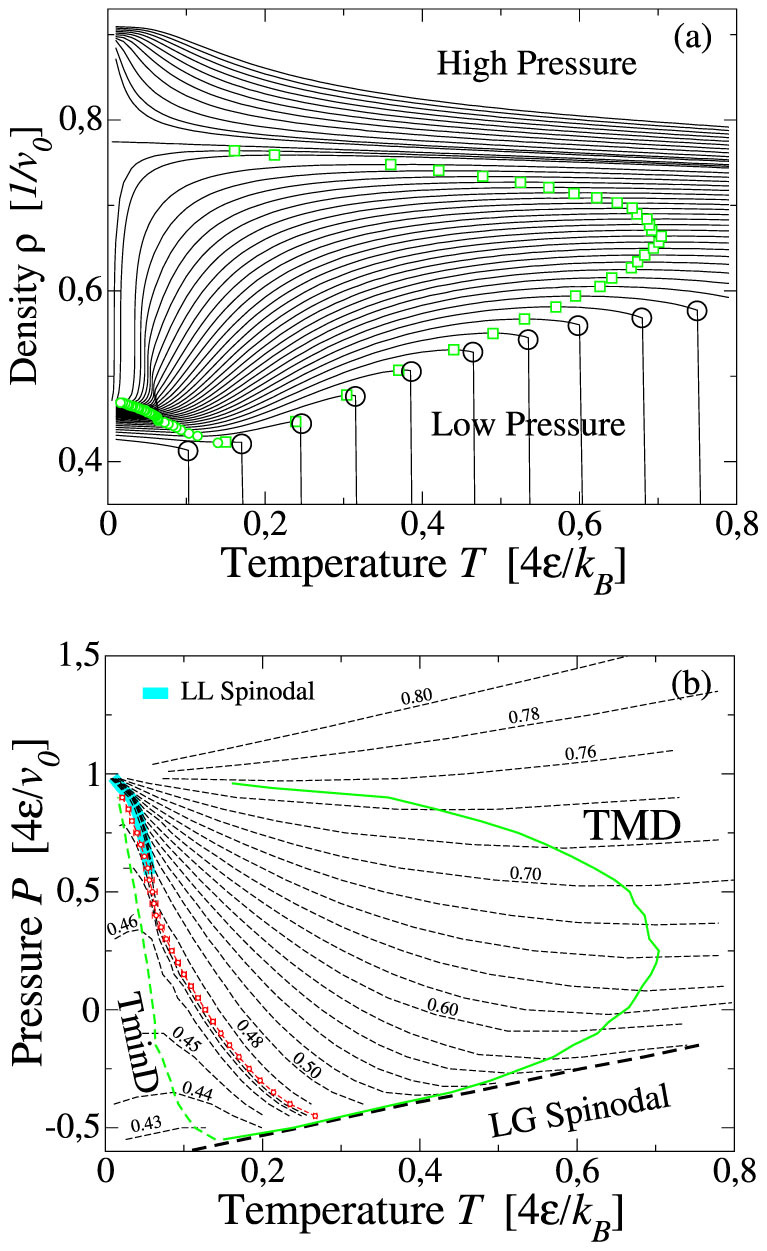
(a) Isobaric density variation for 10^4^ water molecule. Lines join simulated state points (~ 150 for each isobar). *P* increases from −0.5 (bottom curve) to 1.5 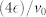 (top curve). Along each isobar we locate the maximum *ρ* (green squares at high *T*) and the minimum *ρ* (green small circles at low *T*) and the liquid-gas spinodal (open large circles at low *P*). (b) Loci of TMD, TminD, liquid-gas spinodal and liquid-liquid spinodal in *T* − *P* plane. Dashed lines with labels represent the isochores of the system from *ρv*_0_ = 0.43 (bottom) to *ρv*_0_ = 0.80 (top). Dashed lines without labels represent intermediate isochores. TMD and TminD correspond to the loci of minima and maxima, respectively, along isochores in the *T* − *P* plane. We estimate the critical isochore at *ρv*_0_ ~ 0.47 (red circles). All the isochores with 0.47 < *ρv*_0_ < 0.76 intersect with the critical isochore for 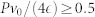 along the LL spinodal (tick turquoise) line.

We calculate the density *ρ* ≡ *N*/*V* of the system as function of *T* along isobars. For a broad range of *P*, we find a maximum and a minimum of density along each isobar (Fig. 2a) according to experimental evidences for bulk and confined water^52^. These maxima and minima identify, for each *P*, the temperature of maximum density (TMD) and the temperature of minimum density (TminD). The TMD locus merges the TminD line as in experiments^52^ and other models^53^.

At low *T* a discontinuous change in *ρ* is observed for 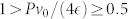, where the parameters *v*_0_ and 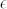 are explained in the Methods section, as it would be expected in correspondence of the HDL-LDL phase transition. At very high pressures (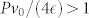) the system behaves as a normal liquid, with monotonically increase of *ρ* upon decrease of *T*.

We estimate the liquid-to-gas (LG) spinodal at 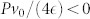 for low *T* (Fig. 2) as the temperature along an isobar at which we find a discontinuous jump of *ρ* to zero value by heating the system. The LG spinodal identifies the locus of the stability limit of liquid phase with respect to the gas phase: at pressures below the LG spinodal in the *P* − *T* plane is no longer possible to equilibrate the system in the liquid phase. The LG spinodal continues at positive pressures ending in the LG critical point (data not shown). We observe that the TMD line approaches the LG spinodal, without touching it (Fig. 2). We recover the LG spinodal also as envelope of isochores (Fig. 2b).

We find a second envelope of isochores at lower *T* and higher *P*, pointing out the liquid-to-liquid (LL) spinodal. Indeed, the two spinodals associated to the LLPT, i.e. the HDL-to-LDL spinodal and the LDL-to-HDL spinodal, collapse one on top of the other and are indistinguishable within our numerical resolution. Nevertheless, we clearly see that isochores are gathering around the points (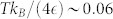, 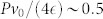) and (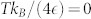, 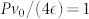), where *k_B_* is the Boltzmann constant, marking two possible critical regions (Fig. 2b).

**Figure 3:**
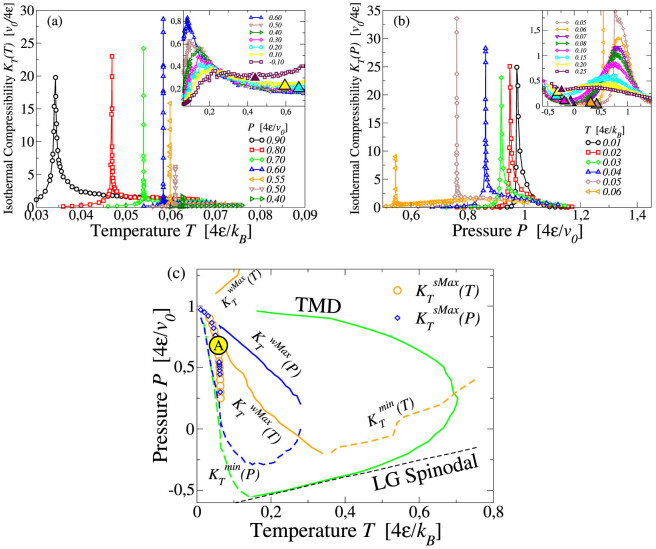
(a) Loci of strong maxima (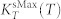), weak maxima (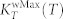 in the inset) and minima (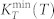 marked with large triangles in the inset) along isobars for *K_T_*(*T*). (b) Loci of strong maxima (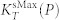), weak maxima (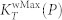 in the inset) and minima (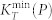 marked with large triangles in the inset) along isotherms. The weak maxima merge with minima. (c) Projection of extrema of *K_T_* in *T* − *P* plane. The strong maxima (symbols), weak maxima (solid lines) and minima (dashed lines) of *K_T_*(*T*) (orange) and *K_T_*(*P*) (blue) form loci in *T* − *P* plane that relate to each other and intersect with the TMD line following the thermodynamic relations discussed in the text. The large yellow circle with label A identifies the region where 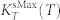 and 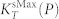 converge and display the largest maxima, consistent with the occurrence of a critical point in a finite-size system. Symbols not explained here are as in Fig. 2.

We calculate the isothermal compressibility by its definition *K_T_* ≡ −(1/〈*V*〉) (∂〈*V*〉/∂*P*)*_T_* and by the fluctuation-dissipation theorem *K_T_* = 〈Δ*V*^2^〉/*k_B_TV* along isobars, *K_T_*(*T*), and along isotherms, *K_T_*(*P*) (Fig. 3), where 〈*V*〉 ≡ *V* is the average volume and 〈Δ*V*^2^〉 the volume fluctuations. We find two loci of extrema for each quantity *K_T_*(*T*) and *K_T_*(*P*): one of strong maxima and one of weak maxima. The loci of strong maxima in *K_T_*(*T*) and *K_T_*(*P*), respectively 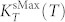 and 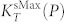, overlap within the error bar with the LL spinodal. The maxima 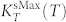 and 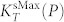 increase in the range of 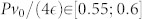 and 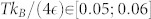 (Fig. 3), consistent with the existence of a critical region. The stronger maxima disappear for 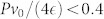.

We find also loci of weak maxima, 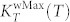 and 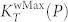 and minima 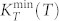 and 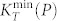. The loci of weak extrema and minima of *K_T_*(*T*) and *K_T_*(*P*) do not coincide in the *T* − *P* plane. The locus of weak maxima along isotherms 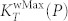 merges with the locus of minima 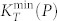 at the point where the slope of both loci is ∂*P*/∂*T* → ∞. Furthermore, both loci approach to the LL spinodal at high *P*. The locus of weak maxima along isobars 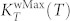 approaches the LL spinodal where *K_T_* exhibits the strongest maxima, and merges with the locus of minima 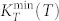 where the slope of both loci is ∂*P*/∂*T* → 0 (data at high *P* and *T* not shown in Fig 3). This locus intersects the TMD at its turning point. Indeed, as reported in Ref. 39 and in the Methods section, the temperature derivative of isobaric *K_T_* along the TMD line is related to the slope of TMD line 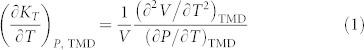
where all the quantities are calculated along the TMD line. Hence the locus of extrema in *K_T_*(*T*), where (∂*K_T_*/∂*T*)*_P_* = 0, crosses the TMD line where the slope (∂*P*/∂*T*)_TMD_ is infinite. We observe also that the weak maxima of *K_T_*(*T*) and *K_T_*(*P*) increase as they approach the LL spinodal. All loci of extrema in *K_T_* are summarized in Fig. 3.

**Figure 4:**
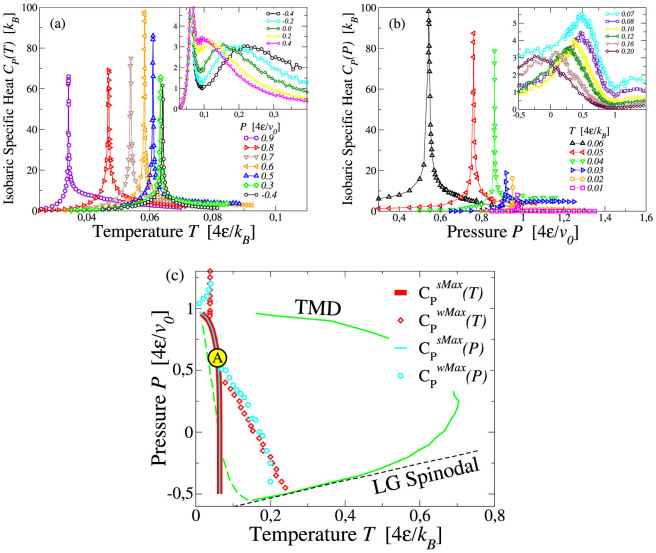
(a) Loci of strong maxima (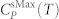) and weak maxima (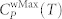 in the inset) along isobars for *C_P_*. (b) Loci of strong maxima (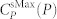) and weak maxima (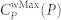 in the inset) along isotherms. (c) Projection of *C_P_* maxima in *T* − *P* plane. The large circle with A identifies the region where *C_P_* shows the strongest maximum. Symbols not explained here are as in Fig. 2.

Next we calculate the isobaric specific heat *C_P_* ≡ (∂〈*H*〉/∂*T*)*_P_* = 〈Δ*H*^2^〉/*k_B_T* along isotherms and isobars, where 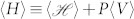 is the average enthalpy, 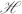 is the Hamiltonian as defined in the Methods section, 〈Δ*H*^2^〉 is the enthalpy fluctuations (Fig. 4). We find two maxima at low *P* separated by a minimum. At high-*T* the maxima are broader and weaker than those at low-*T*. As discussed in Ref. 49, the maxima at high *T* are related to maxima in fluctuations of the HB number *N*_HB_, while the maxima at low *T* are a consequence of maxima in fluctuations of the number *N*_coop_ of cooperative HBs. The lines of strong maxima at constant *P* and constant *T*, respectively 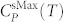 and 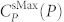, overlap for all the considered pressures, and both maxima are more pronounced in the range 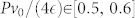 and 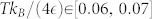. The weak maxima 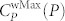 and 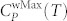 increase approaching the LL spinodal and have their larger maxima at the state point where they converge to the strong maxima, consistent with the occurrence of a critical point for a finite system (Fig. 4). The lines of weak maxima overlap for all positive pressures, branching off at negative pressures. At negative pressures, the locus 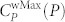 bends toward the turning point of the TMD line, as discussed in Methods section and in Ref. 53. Indeed, according to the relation 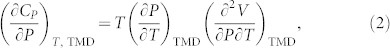
in case of intersection between the locus of extrema (∂*C_P_*/∂*P*)*_T_* = 0 and the TMD line, it results that (∂*P*/∂*T*)_TMD_ = 0. Note that, as we explain in the Methods section, the relation (2) does not imply any change in the slope of the TminD line at the intersection with the locus of (∂*C_P_*/∂*P*)*_T_* = 0.

**Figure 5:**
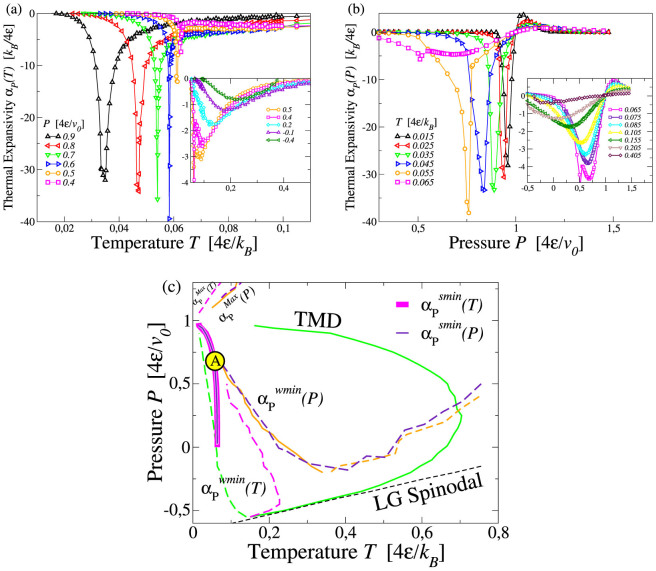
(a) Loci of strong minima of (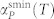) and weak minima (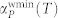 in the inset) along isobars for *α_P_*. (b) Loci of strong minima (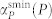) and weak extrema (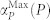 and 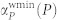 in the inset) along isotherms. (c) Projection of *α_P_* extrema in *T* − *P* plane. Orange lines are the loci of weaker extrema 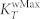 and 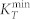. The large circle with A identifies the region where the divergent minimum in *α_P_* is observed. Symbols not explained here are as in Fig. 2.

We calculate also the thermal expansivity *α_P_* ≡ (1/〈*V*〉) (∂〈*V*〉/∂*T*)*_P_* along isotherms and isobars (Fig. 5). As for the other response functions, we find two loci of strong extrema, minima in this case, 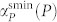 and 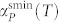, along isotherms and isobars, respectively showing a divergent behavior in the same region where we find the strong maxima of *K_T_* and *C_P_*. From this region two loci of weaker minima depart. We find that the locus of weak minima along isobars 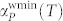 bends toward the turning point of the TMD. Although our calculations for *α_P_* do not allow us to observe the crossing with the TMD line, based on the relation (see Methods) 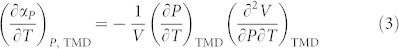
that holds at the TMD line, we can conclude that 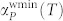 should have zero *T*-derivative if it crosses the point where the TMD turns into the TminD line, because in this point the TMD slope approaches zero.

The locus of weaker minima along isotherms 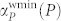, merges with the locus of maxima 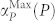 at the state point where the slope of both loci is ∂*P*/∂*T* → ∞ (not shown in Fig. 5). According to the thermodynamic relation, discussed in Methods section, 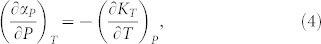
we find that the locus of extrema in thermal expansivity along isotherms coincides, within the error bars, with the locus of extrema of isothermal compressibility along isobars (Fig. 5c).

All the loci of extrema of response functions that converge toward the same region *A* in Fig. 3, 4 and 5 increase in their absolute values. Because the increase of response functions is related to the increase of fluctuations and this is, in turn, related to the increase of correlation length *ξ*, to estimate *ξ* we calculate the spatial correlation function 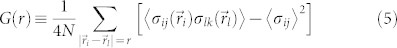
where 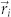 is the position of the molecule *i*, 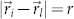 the distance between molecule *i* and molecule *l* and 〈·〉 the thermodynamic average. The states of the water molecule, as well as the density *ρ*, the energy *E* and the entropy *S* of the system, are completely described by the bonding variables *σ_ij_*. Therefore, the function *G*(*r*) accounts for the fluctuations in *ρ*, *E* and *S* and allows us to evaluate the correlation length because the order parameter of the LLPT, as we discuss in the following, is related to a linear combination of *ρ* and *E*. Note that, instead, the density-density correlation function would give only an approximate estimate of *ξ*.

We observe an exponential decay of *G*(*r*) ~ *e*^−*r*/*ξ*^ at high temperatures in a broad range of pressures. Approaching the region *A*, the correlation function can be written as *G*(*r*) ~ *e*^−*r*/*ξ*^/*r^d^*^−2+*η*^ where *d* is the dimension of the system and *η* a (critical) positive exponent. When *ξ* is of the order of the system size, the exponential factor approaches a constant leaving the power-law as the dominant contribution for the decay.

**Figure 6:**
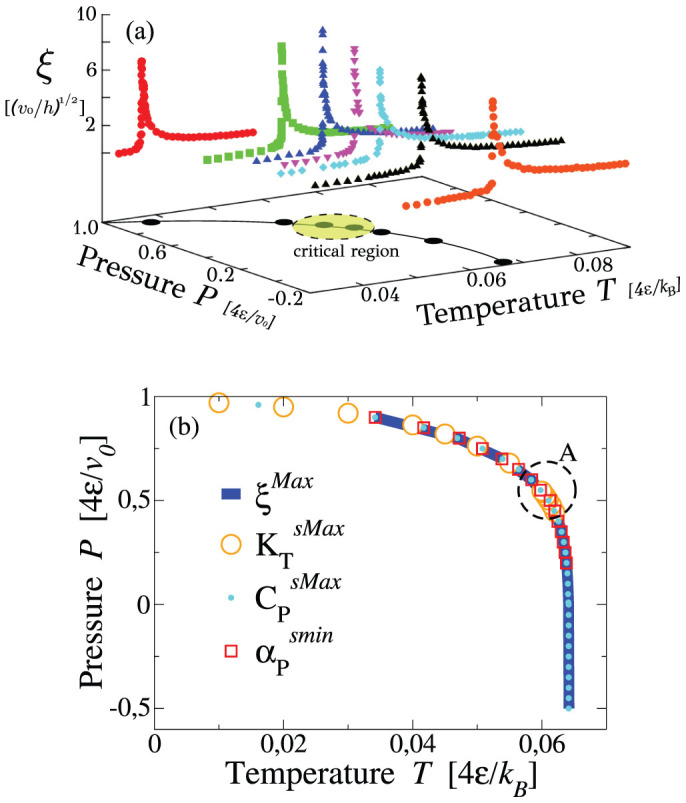
(a) The correlation length *ξ* along isobars for *N* = 10^4^ water molecules has maxima that increase for *P* approaching the critical region A. (b) The locus of *ξ* maxima coincides with the loci of strong extrema of *K_T_*, *C_P_* and *α_P_*. The Widom line is by definition the locus of *ξ* maxima at high *T* departing from the LLCP, that we locate within the critical region A, as discussed in the text.

At *P* below the region *A*, we find that *ξ* has a maximum, *ξ*^Max^, along isobars and that *ξ*^Max^ increases approaching *A* (Fig. 6). The *ξ*^Max^ locus coincides with the locus of strong extrema of *C_P_*, *K_T_* and *α_P_* (Fig. 6b). We observe that this common locus converges to *A* and that all the extrema increase approaching *A*. This behavior is consistent with the identification of *A* with the critical region of the LLCP. Furthermore, we identify the common locus with the Widom line that, by definition, is the *ξ*^Max^ locus departing from the LLCP in the one-phase region^54^,^55^. Our calculations allow us to locate the Widom line at any *P* down to the liquid-to-gas spinodal.

At *P* above the region *A*, we find the continuation of the *ξ*^Max^ line, but with maxima that decrease for increasing *P*, as expected at the LL spinodal that ends in the LLCP (Fig. 6). Therefore, we identify the high-*P* part of the *ξ*^Max^ locus with the LL spinodal. Along this line the density, the energy and the entropy of the liquid are discontinuous, as discussed in previous works^31^,^40^,^44^,^45^,^46^,^47^,^48^,^49^.

To better locate and characterize the LLCP in *A* we need to define the correct order parameter (o.p.) describing the LLPT. According to mixed-field finite-size scaling theory^56^, a density-driven fluid-fluid phase transition is described by an o.p. *M* ≡ ρ* + su*, where ρ* = *ρv*_0_ represents the leading term (number density), 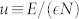 is the energy density (both quantities are dimensionless) and *s* is the mixed-field parameter. Such linear combination is necessary in order to get the right symmetry of the o.p. distribution *Q_N_*(*M*) at the critical point where 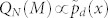. Here is *x* ≡ *B*(*M* − *M_c_*), 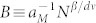, *β* is the critical exponent that governs *M*, *ν* is the critical exponent that governs *ξ*, with *ν* and β defined by the universality class, *a_M_* is a non-universal system-dependent parameter and 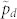 is an universal function characteristic of the Ising fixed–point in *d* dimensions. We adjust *B* and *M_c_* so that *Q_N_*(*M*) has zero mean and unit variance.

**Figure 7:**
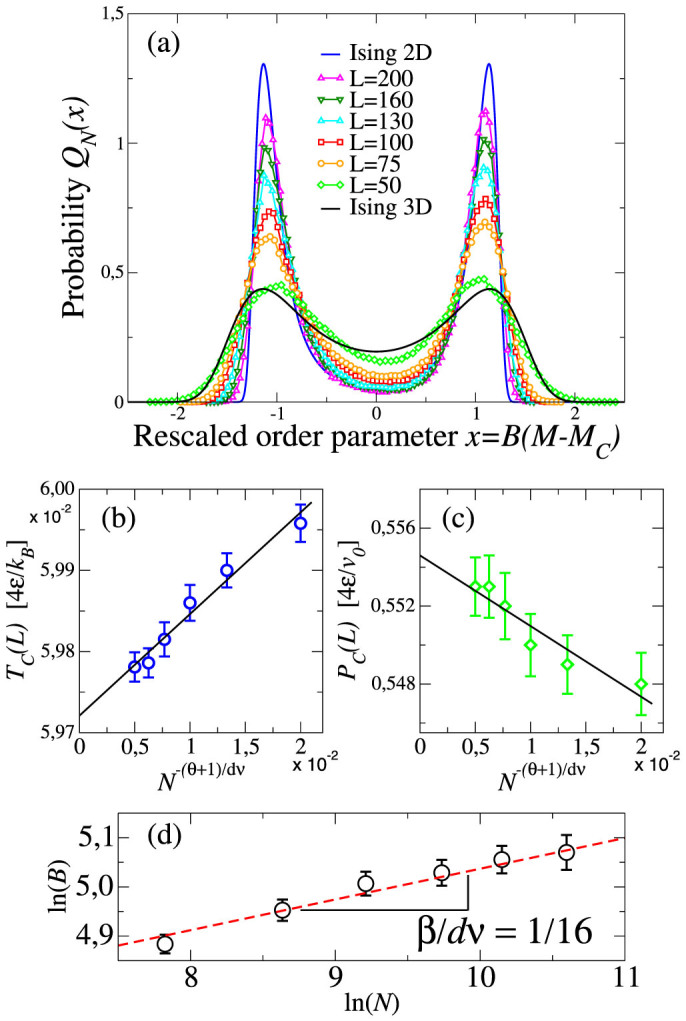
(a) The size-dependent probability distribution *Q_N_* for the rescaled o.p. *x*, calculated for *T_c_*(*N*), *P_c_*(*N*) and *B*(*N*), has a symmetric shape that approaches continuously (from *N* = 2500, symbols at the top at *x* = 0, to *N* = 40000, symbols at the bottom) the limiting form for the 2D Ising universality class (full blue line) and differs from the 3D Ising universality class case (full black line). Error bars are smaller than the symbols size. (b) The size-dependent LLCP temperature *T_c_*(*N*) and (c) pressure *P_c_*(*N*) (symbols), resulting from our best-fit of *Q_N_*, extrapolate to 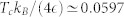 and 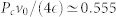, respectively, following the expected linear behaviors (lines). (d) The normalization factor *B*(*N*) (symbols) follows the power law function (dashed line) ∝ *N^β/dν^*. We use the *d* = 2 Ising critical exponents: *θ* = 2 (correction to scaling), *ν* = 1 and *β* = 1/8 (both defined in the text).

We combine, using the multiple histogram reweighting method^57^ described in the Methods section, a set of 3 × 10^4^ MC independent configurations for ~ 300 state points with 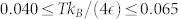 and 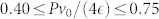. We verify, by tuning *s*, *T* and *P*, that there is a point within the region *A* where the calculated *Q_N_*(*x*) has a symmetric shape with respect to *x* = 0 (Fig. 7). We find *s* = 0.25 ± 0.03 for our range of *N*. The resulting critical parameters *T_c_*(*N*), *P_c_*(*N*) and the normalization factor *B*(*N*) follow the expected finite-size behaviors with 2D Ising critical exponents^56^. From the finite-size analysis we extract the asymptotic values 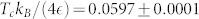 and 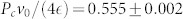.

**Figure 8:**
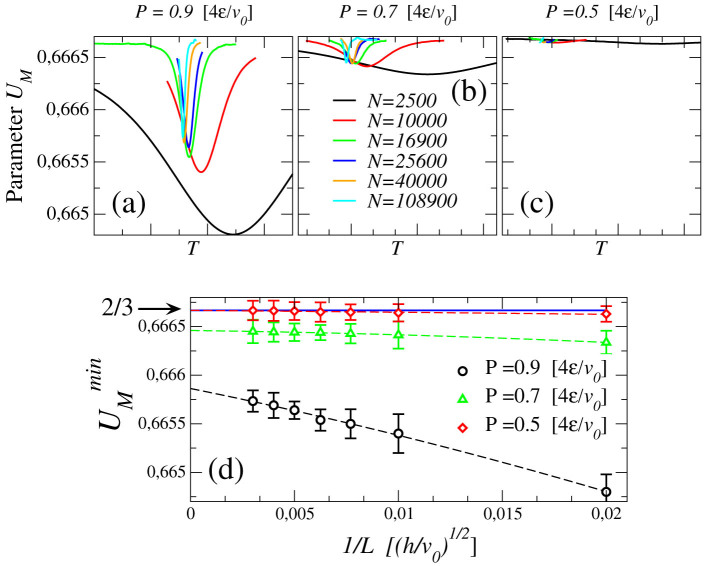
Challa-Landau-Binder parameter *U_M_* (defined in the text) of the o.p. *M* for different system sizes, calculated for three pressures: (a) 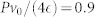, (b) 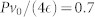, and (c) 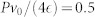 slightly below 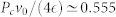. The curves are calculated with the histogram reweighting method. (d) Scaling of the minima of *U_M_* for different *P*. The arrow points to value 2/3 corresponding to the absence of a first-order phase transition in the thermodynamic limit. Error bars are calculated propagating the statistical error from histogram reweighting method.

The presence of a first order phase transition ending in a critical point, associated to the o.p. *M*, is confirmed by the finite size analysis of the Challa-Landau-Binder parameter^58^ of *M*

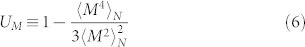
where the symbol 〈·〉*_N_* refers to the thermodynamic average for a system with *N* water molecules. *U_M_* quantifies the bimodality in *Q_N_*(*M*). The isobaric value of *U_M_* shows a minimum at the temperature where *Q_N_*(*M*) mostly deviates with respect to a symmetric distribution (Fig. 8). Minimum of *U_M_* converges to 2/3 in the thermodynamic limit away from a first order phase transition, while it approaches to a value <2/3 where the bimodality of *Q_N_*(*M*) indicates the presence of phase coexistence.

**Figure 9:**
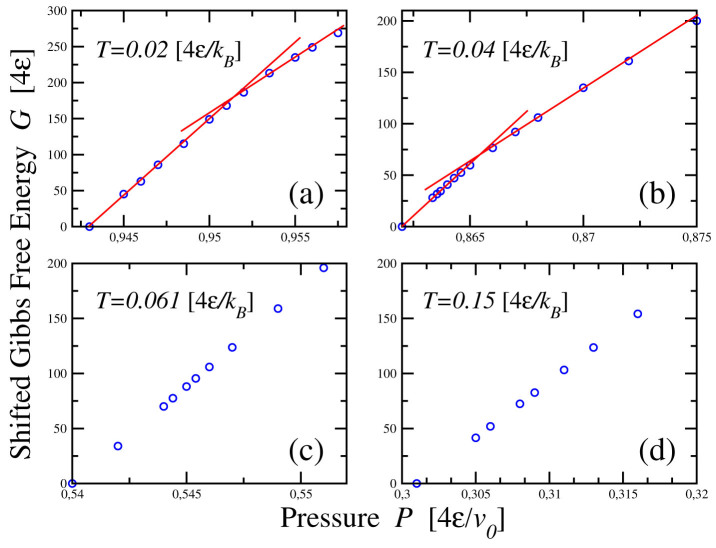
Gibbs free energy *G* along isotherms, as function of *P*. Points are shifted so that *G* = 0 at the lowest *P*. Lines are guides for the eyes. (a) For 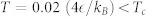 there is a discontinuity in the *P*-derivative of *G* at 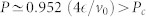 as expected at the LLPT, consistent with the behavior of the response functions at this state point (e.g., in Fig. 3b, 4b). (b) For 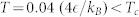 we observe the discontinuity in the *P*-derivative at 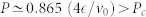, again consistent with the LLPT. The LDL has a lower chemical potential (*μ* ≡ *G*/*N*) than the HDL, *μ_LDL_* < *μ_HDL_*, due to the HB energy gain in the LDL. For 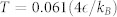 (c) and for 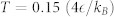 (d), both larger than *T_c_*, we instead do not observe any discontinuity in the *P*-derivative of *G* by crossing the locus of 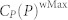 and the locus of 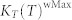, respectively, as expected in the one-phase region.

These results are consistent with the behavior of the Gibbs free energy *G* calculated with the histogram reweighting method (Fig. 9). In particular, we calculate *G* along isotherms, for *P* crossing the LLPT and the loci of weak maxima in *K_T_*(*T*) and *C_P_*(*P*). We find that the behavior of *G* for *T* < *T_c_* is consistent with the occurrence of a discontinuity in volume *V* = ∂*G*/∂*P*, in the thermodynamic limit, with a decrease of *V* corresponding to the transition from LDL to HDL for increasing *P*. Crossing the loci 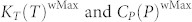 the volume decreases with pressure without any discontinuity as expected in the one-phase region.

**Figure 10:**
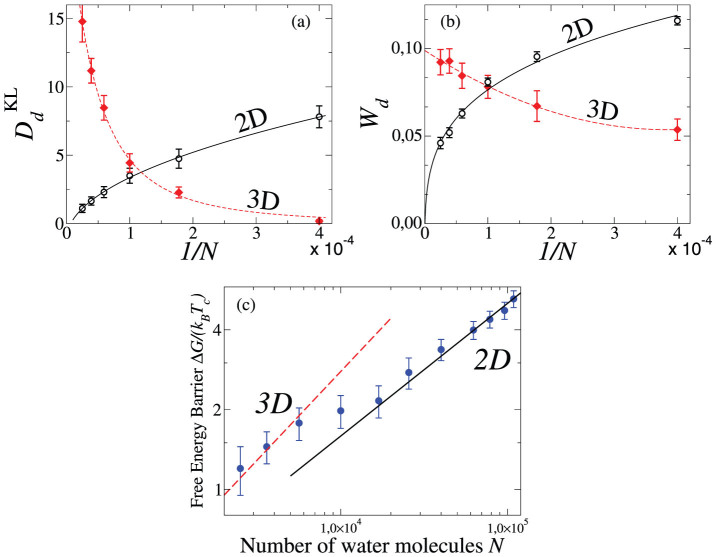
(a) Kullback-Leibler divergence 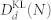 and (b) Liu et al. deviations *W_d_* of the calculated 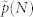 from the Ising universal function 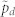 in *d* = 2 (open symbols) and *d* = 3 (closed symbols), as a function of 1/*N*, with *N* water molecules, at constant 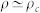. In both panels lines are power-law fits and we observe a crossover between 2D and 3D behavior at 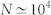. (c) The free-energy cost to form an interface between the two liquids coexisting at the LLCP scales as 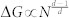 with *d* = 3 for *N* < 10^4^ and *d* = 2 for *N* > 10^4^.

The distribution *Q_N_*(*N*) adjust well to the data only for large *N*. We, therefore, perform a more systematic analysis. For each *N*, we quantify the deviation of the calculated 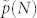 from the expected 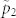 for the 2D Ising. Furthermore, due to the behavior of data for small *N* (Fig. 7a), we calculate the deviation from the 3D Ising 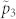
56. We estimate the Kullback-Leibler divergence^51^,^59^, 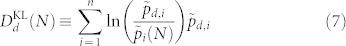
of the probability distribution 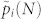 of *x_i_* from the theoretical value 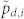 of *x_i_* (*i* = 1, …, *n*) in *d* dimensions (Fig. 10a), and the Liu et al. deviation^51^, 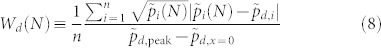
with 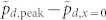 difference between the distribution peak and its value at *x* = 0 (Fig. 10b).

We confirm 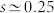 for 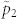 and find *s* = 0.10 ± 0.02 for 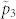 for our range of *N*. For both 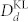 and *W_d_*, with *d* = 2 and *d* = 3, we find minima at 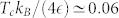 and 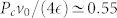 that become stronger for increasing *N*. We find that 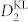 and *W*_2_ decrease with increasing *N*, vanishing for *N* → ∞ (Fig. 10). Therefore, for an infinite monolayer between hydrophobic walls separated by *h* ≈ 0.5 nm, the system has a LLCP that belongs to the 2D Ising universality class, as expected from our representation of the system as the 2D projection of the monolayer.

However, by increasing the confinement, i.e. reducing *N* and *L* at constant *ρ*, 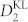 and *W*_2_ become larger than 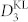 and *W*_3_, respectively. Therefore, the calculated 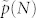 deviates from the 3D probability distribution less than from the 2D probability distribution. For *N* = 2500 we find that both 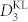 and *W*_3_ have values approximately equal to those for 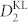 and *W*_2_ calculated for a system ten times larger. In particular we find 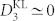 for *N* = 2500. Hence, by increasing the confinement of the monolayer at constant *ρ*, the LLCP has a behavior that approximates better the bulk^25^,^26^,^27^,^28^,^29^,^30^,^38^, with a crossover between 2D and 3D-behavior occurring at 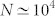.

This dimensional crossover is confirmed by the finite-size analysis of the Gibbs free energy cost Δ*G*/(*k_B_T_c_*) to form an interface between the two liquids in the vicinity of the LLCP, calculated as 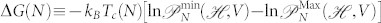, where 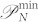 and 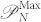 are the minimum and maximum values of the probability distribution 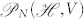 of configurations of *N* water molecules with energy 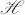 and volume *V* at the LLCP. This quantity is expected to scale as 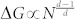. We find that our data can be fitted as 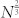 for small sizes and as 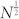 for large sizes with a crossover around *N* = 10^4^ (Fig. 10c). Considering the value of the estimated *ρ_c_* in real units (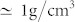)^45^, the corresponding crossover wall-size is 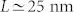.

## Discussion

Our rationale for this dimensional crossover at fixed *h* is that, when *L*/*h* decreases toward 1, the characteristic way the critical fluctuations spread over the system, i.e. the universality class of the LLCP, resembles closely the bulk because the asymmetry among the three spatial dimensions is reduced. A similar result was found recently by Liu et al. for the gas-liquid critical point of a Lennard-Jones (LJ) system confined between walls by fixing *L* and varying *h*^51^. However, in the case considered by Liu et al. the crossover was expected because the number of layers of particles was increased from one to several, making the system more similar to the isotropic 3D case. Here, instead, we consider always one single layer, changing the proportion *L*/*h* by varying *L*. Therefore, it could be expected that the system belongs to the 2D universality class for any *L*.

Furthermore, the extrapolation of the results for the LJ liquid to our case of a monolayer with 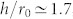, where *r*_0_ is the water van der Waals diameter, would predict a dimensional crossover at 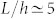
51. Here, instead, we find the crossover at 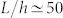, i.e. one order of magnitude larger than the LJ case. We ascribe this enhancement of the crossover to (i) the presence of a cooperative HB network and (ii) the low coordination number that water has in both the monolayer and the bulk. These are the main differences between water and a LJ fluid. The cooperativity intensifies drastically the spreading of the critical fluctuations along a network, contributing to the effective dimensionality increase of the confined monolayer. Moreover, the HB network has in 3D a coordination number (*z* = 4) as low as in 2D, making the first coordination shell similar in both dimensions.

Our findings are consistent with recent atomistic simulations of water nanoconfined between surfaces.^60^,^61^,^62^. Zhang et al. found that water dipolar fluctuations are enhanced in the direction parallel to the confining surfaces (hydrophobic graphene sheets) within a distance of 0.5 nm^60^. Ballenegger and Hansen found similar results for confined polar fluids, including water, within ≈ 0.5 nm distance from the hydrophobic surface^61^. Bonthuis et al. extended these results to both hydrophilic and hydrophobic confining surfaces. All these findings are consistent with our result showing the enhancement of the fluctuations of the o.p. in the direction parallel to the confining walls separated by *h* ≈ 0.5 nm. Furthermore, Zhang et al. observed that the effect does not depend on the details of the water-surface interaction but stems from the very presence of interfaces^60^. This is confirmed by our study, where the water-interface interaction is purely due to excluded volume. Following the authors of Ref. 60, this observation allows us to relate our finding for rigid surfaces to experimental results for water hydrating membranes^63^, reporting new types of water dynamics in thin interfacial layers, and water nanoconfined in different types of reverse micelles^64^, showing that the water dynamics is governed by the presence of the interface rather than the details (e.g., the presence charged groups) of the interface.

In conclusion, we analyze the low-*T* phase diagram of a water monolayer confined between hydrophobic parallel walls of size *L* separated by *h* ≈ 0.5 nm. We study water fluctuations associated to the thermodynamic response functions and their relations to the loci of TMD, TminD. For each response function we find two loci of extrema, one stronger at lower-*T* and one weaker and broader at higher-*T*. These loci converge toward a critical region where the fluctuations diverge in the thermodynamic limit, defining the LLCP. We calculate the Widom line departing from the LLCP based on its definition as the locus of maxima of *ξ* and show that it coincides with the locus of strong maxima of the response functions. We find that the LLCP belongs to the 2D Ising universality class for *L* → ∞, with strong finite-size effects for small *L*. Surprisingly, the finite-size effects induce the LLCP universality class to converge toward the bulk case (3D Ising universality class) already for a system with a very pronounced plane asymmetry, i.e. a water monolayer of height *h* ≈ 0.5 nm and *L*/*h* ≈ 50. For normal liquid, instead, this is expected only for much smaller relative values of *L* (*L*/*h* ≤ 5). We rationalize this result as a consequence of two properties of the HB network: (i) its high cooperativity, that enhances the fluctuations, and (ii) its low coordination number, that makes the first coordination shell for the monolayer and the bulk similar.

## Methods

### The model

We consider a monolayer formed by *N* water molecules confined in a volume *V* ≡ *hL*^2^ between two hydrophobic flat surfaces separated by a distance *h*, with 
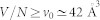
, where *v*_0_ is the water excluded volume. Each water molecule has four next-neighbours^7^. We partition the volume into *N* equivalent cells of height 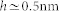 and square section with size 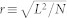, equal to the average distance between water molecules. By coarse-graining the molecules distance from the surfaces, we reduce our monolayer representation to a 2D system. We use periodic boundary conditions parallel to the walls to reduce finite-size effects. We simulate constant *N*, *P*, *T*, allowing *V*(*T*, *P*) to change, with each cell *i* = 1, …, *N* having number density 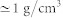. To each cell we associate a variable *n_i_* = 0 (*n_i_* = 1) depending if the cell *i* has *ρ_i_*/*ρ*_0_ ≤ 0.5 (*ρ_i_*/*ρ*_0_ > 0.5). Hence, *n_i_* is a discretized density field replacing the water translational degrees of freedom. The water-water interaction is given by 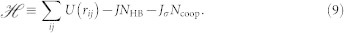
The first term, summed over all the water molecules *i* and *j* at O–O distance *r_ij_*, has *U*(*r*) ≡ ∞ for 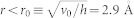 (water van der Waals diameter), 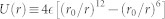 for *r* ≥ *r*_0_ with 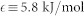, and *U*(*r*) ≡ 0 for *r* > *r_c_* ≡ 25*r*_0_ (cutoff).

The second term represents the directional (covalent) component of the hydrogen bond (HB), with 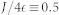, 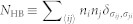 number of HBs, with the sum over n.n., where *σ_ij_* = 1, …, *q* is the bonding index of molecule *i* to the n.n. molecule *j*, with *δ_ab_* = 1 if *a* = *b*, 0 otherwise. Each water molecule can form up to four HBs. We adopt a geometrical definition of the HB, based on the 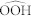 angle and the OH—O distance. A HB breaks if 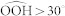. Hence, only 1/6 of the entire range of values [0, 360°] for the 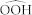 angle is associated to a bonded state. Therefore, we choose *q* = 6 to account correctly for the entropy variation due to the HB formation and breaking. Moreover, a HB breaks when the OH—O distance > *r*_max_ − *r*_OH_ = 3.14 Å, where *r*_OH_ = 0.96 Å and *r*_max_ = 4.1 Å. The value of *r*_max_ is a consequence of our choice *n_i_* = 0 for *ρ_i_*/*ρ*_0_ ≤ 0.5, i.e. 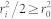, implying that *n_i_n_j_* = 0 when 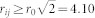 Å ≡ *r*_max_.

The third term of the Eq.(9) accounts for the HB cooperativity due to the quantum many-body interaction^65^, with 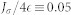 and 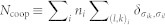, where (*l*, *k*)*_i_* indicates each of the six different pairs of the four indices *σ_ij_* of a molecule *i*. The value 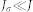 is chosen in such a way to guarantee an asymmetry between the two components of the HB interaction. To the cooperative term is due the O–O–O correlation that locally leads the molecules toward an ordered configuration. In bulk water this term would lead to a tetrahedral structure at low *P* up to the second shell, as observed in the experiments^66^. An increase of *T* or *P* partially disrupts the HB network and induces a more compact local structure, with smaller average volume per molecule. Therefore, for each HB we include an enthalpy increase *Pv*_HB_, where *v*_HB_/*v*_0_ = 0.5 is the average volume increase between high-*ρ* ices VI and VIII and low-*ρ* (tetrahedral) ice Ih. Hence, the total volume is *V* ≡ *V*_0_ + *N*_HB_*v*_HB_, where *V*_0_ ≥ *Nv*_0_ is a stochastic continuous variable changing with Monte Carlo (MC) acceptance rule^46^. Because the HBs do not affect the n.n. distance^66^, we ignore their negligible effect on the *U*(*r*) term. Finally, we model the water-wall interaction by excluded volume.

### The observables

The LLCP is identified by the mixed-field order parameter *M* and not by the magnetization of the Potts variables *σ_i_*_,*j*_ as in normal Potts model. *M* is related to the configuration of the system by the relation 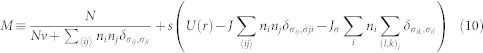
where *v* ≡ *V*_0_/*N* and *s* is the mixed-field parameter. *M* is therefore a linear combination of density and energy.

Thermodynamic response functions are calculated from 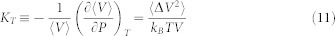
and 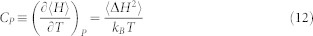
as long as the volume and energy distributions are not clearly bimodal, i.e. excluding the values of *T* and *P* where the phase coexistence is observed, based on the definition of *M*. Here 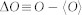, for 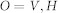 and, *H* is the enthalpy of the system.

### The Monte Carlo method

The system is equilibrated via Monte Carlo simulation with Wolff algorithm^46^, following an annealing procedure: starting with random initial condition at high *T*, the temperature is slowly decreased and the system is re-equilibrated and sampled with 10^4^ ÷ 10^5^ independent configurations for each state point. The thermodynamic equilibrium is checked probing that the fluctuation-dissipation relations, Eq. (11) and (12), hold within the error bar.

### The histogram reweighting method

The probability *Q_N_*(*M*) is calculated in a continuous range of *T* and *P* across the *ξ*^Max^ line. We consider an initial set of *m* ∈ [10:20] independent simulations within a temperature range 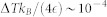 and a pressure range 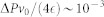. For each simulation *i* = 1, …, *m* we calculate the histograms *h_i_*(*u*, *ρ*) in the energy density–density plane. The histograms *h_i_*(*u*, *ρ*) provide an estimate of the equilibrium probability distribution for *u* and *ρ*; this estimate becomes correct in the thermodynamic limit. For the *NPT* ensemble, the new histogram *h*(*u*, *ρ*, *P*′, *β*′) for new values of *β*′ = 1/*k_B_T*′ and *P*′ close the simulated ones, is given by the relation^57^

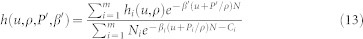
where *N_i_* is the number of independent configurations of the run *i*. The constants *C_i_*, related to the Gibbs free energy value at *T_i_* and *P_i_*, are self-consistently calculated from the equation^57^

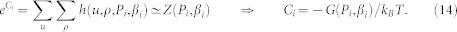
We choose as initial set of parameters *C_i_* = 0. The parameters *C_i_* are recursively calculated by means of Eq. (13) and (14) until the difference between the values at iteration *k* and *k* + 1 is less then the desired numerical resolution (10^−3^ in our calculations). Once the new histogram is calculated, *Q_N_*(*M*) at *T_i_* and *P_i_* is calculated integrating *h*(*u*, *ρ*, *P_i_*, *β_i_*) along a direction perpendicular to the line *ρ* + *su*.

### Thermodynamic relations

We report here the calculations for the thermodynamic relations in Eq. (1), (2), (3) and (4)^39^. To verify the relation (4) we calculate the derivative of *K_T_* along isobars 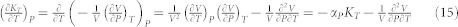
and the derivative of *α_P_* along isotherms 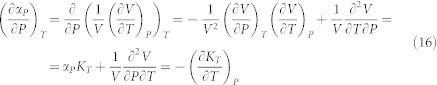
Following^39^,^67^ the line of extrema in density (TMD and TminD lines) is characterized by *α_P_* = 0, hence, *dα_P_* = 0 along the TMD line. Therefore, 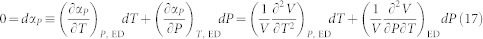
where the index “ED” denotes that the derivatives are taken along the locus of extrema in density. So, the slope ∂*P*/∂*T* of TMD is given by 
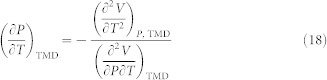
from which, using Eq. (15) with *α_P_* = 0, we get Eq. (1). The Eq. (18) holds as long as both (∂*α_P_*/∂*P*)*_T_* and (∂*α_P_*/∂*T*)*_P_* do not vanish contemporary, as it occurs along the Widom line, where the loci of strong minima of *α_P_* overlap. For this reason the intersection between the Widom line and TminD line does not imply any change in the slope (∂*P*/∂*T*)_TminD_.

To calculate Eq. (2) we start from *C_P_* and *α_P_* written in terms of Gibbs free energy 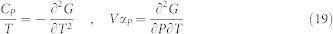
from which results 
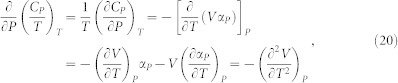

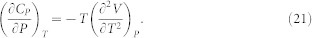


Substituting in Eq. (18) we get the Eq. (2) at the TMD. Moreover, because of *α_P_* = 0 at the TMD line, from the last equivalence of Eq. (20) we get 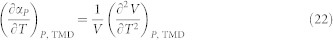
from which, using Eq. (18), we get the Eq. (3).

## Author Contributions

V.B. and G.F. designed the research. V.B. made the simulations. G.F. supervised the work. Both authors analyzed the data, prepared the figures, wrote the text and reviewed the manuscript.

## Figures and Tables

**Figure 1:**
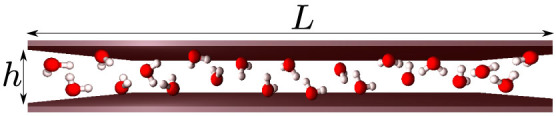
Schematic view of a section of the water monolayer confined between hydrophobic walls of size *L* × *L* separated by *h* ≈ 0.5 nm.
